# Metabolic Signatures Associated with COVID-19 Vaccination in Serum from Healthy Individuals

**DOI:** 10.3390/vaccines14090771

**Published:** 2026-09-02

**Authors:** Mariam M. AlEissa, Refat M. Nimer, Reem H. AlMalki, Randh AlAhmari, Ahdab A. Alsaieedi, Monera Alrukhayes, Nada Saleh, Raef R. Albugami, Raghad A. AlQurashi, Esraa A. Hawsa, Muath Ben Shaded, Afshan Masood, Sami S. Almudarra, Assim A. Alfadda, Hamad H. Alonazi, Abdullah M. Assiri, Anas Abdel Rahman

**Affiliations:** 1 College of Medicine, Alfaisal University, Riyadh 11533, Saudi Arabia; 2 Population Health Observatory, Ministry of Health, Riyadh 11176, Saudi Arabia; 3 Public Health Laboratory, Public Health Authority, Riyadh 11176, Saudi Arabia; 4 King Khaled Eye Specialist Hospital (KKESH) Research Center, Riyadh 11462, Saudi Arabia; 5 Computational Sciences Department, Centre for Genomic Medicine (CGM), King Faisal Specialist Hospital & Research Center, Riyadh 11211, Saudi Arabia; 6 Department of Medical Laboratory Sciences, Faculty of Applied Medical Sciences, Jordan University of Science and Technology, Irbid 22110, Jordan; 7 Metabolomics Section, Precision Medicine Laboratory Department, Genomics Medicine Center of Excellence, King Faisal Specialist Hospital and Research Centre (KFSHRC), Riyadh 11211, Saudi Arabia; 8 Department of Medical Laboratory Sciences, Faculty of Applied Medical Sciences, King Abdulaziz University, Jeddah 21589, Saudi Arabia; 9 Vaccines and Immunotherapy Unit, King Fahd Medical Research Center, King Abdulaziz University, Jeddah 21589, Saudi Arabia; 10 Department of Medical Laboratory Sciences, Faculty of Allied Health Sciences, Health Sciences Center (HSC), Kuwait University, Jabriya 90805, Kuwait; 11 College of Medicine, King Saud Bin Abdulaziz University for Health Sciences (KSAU-HS), Riyadh 11426, Saudi Arabia; salehn@ksau-hs.edu.sa; 12 King Abdullah International Medical Research Centre (KAIMRC), Riyadh 14611, Saudi Arabia; 13 Ministry of National Guard Health Affairs (MNGHA), Riyadh 11426, Saudi Arabia; 14 Cellular Therapy Laboratory, King Fahad Medical City (KFMC), Riyadh Second Healthcare Cluster, Riyadh 11525, Saudi Arabia; 15 Proteomics Resource Unit, Obesity Research Center, College of Medicine, King Saud University, Riyadh 11461, Saudi Arabia; afsmasood@ksu.edu.sa (A.M.); aalfadda@ksu.edu.sa (A.A.A.); 16 Gulf Center for Disease Prevention and Control, Gulf Health Council, Riyadh 7431, Saudi Arabia; 17 Department of Medicine, College of Medicine and King Saud Medical City, King Saud University, Riyadh 11461, Saudi Arabia; 18 Strategic Center for Diabetes Research, College of Medicine, King Saud University, Riyadh 12372, Saudi Arabia; 19 The Clinical Trials Office (CTO), University of Illinois Chicago (UIC), Chicago, IL 60612, USA

**Keywords:** COVID-19 vaccination, metabolomics, eicosanoids, cyclic AMP, kynurenine, dopamine, NAD^+^, dose–response, UPLC-QTOF, systems vaccinology

## Abstract

**Background**: COVID-19 vaccines have proven effective in reducing severe disease and mortality from SARS CoV-2 infection. The underlying molecular mechanisms and alterations in the human serum metabolome influencing the effectiveness and development of immunity remain unclear. **Methods:** Serum samples were collected from 29 healthy individuals at three time points: prior to vaccination (A), post-first dose (B), and post-second dose (C). Untargeted high-resolution (HR) liquid chromatography coupled with mass spectrometry (LC-MS) was performed on these samples. Metabolites showing significant differential abundance at each time point were identified, and both multivariate and univariate statistical analyses were performed to determine changes associated with the pairwise comparisons, priming (A vs. B), booster (B vs. C), and the overall vaccine effect (A vs. C). Vaccination-specific features were determined after excluding metabolites associated with SARS-CoV-2 IgG seropositivity to better isolate vaccine-driven metabolic changes. Bioinformatics, pathway, and network analyses were conducted using Ingenuity Pathway Analysis (IPA) to identify relevant pathways. **Results:** Our study identified significant metabolic changes across the three time points. A total of 377 metabolites were identified, of which 59 metabolites, including prostaglandins, eicosanoids, and lipids, were shared across all three groups. The majority of these metabolites showed an initial decrease after the first dose, followed by broad upregulation after the second dose. We identified 1 (downregulated), 34 (26 upregulated and 8 downregulated), and 18 (2 upregulated and 16 downregulated) unique metabolites in the priming, booster, and the overall vaccine effect groups, respectively. L-3-hydroxykynurenine was observed to be significantly reduced by the priming dose effect. By contrast, the booster effect showed decreased myo-inositol 1,3,4,5-tetrakisphosphate, while levels of DL-DOPA, 3-methoxytyrosine, and prostaglandin-esterified phospholipids, including PC(P-16:0/PGF1α) and PE(PGF1α/18:0), increased. On the other hand, the overall vaccine effect revealed decreased cyclic AMP and increased 3′-O-methyladenosine levels. These changes were associated with perturbations in arachidonic acid metabolism, glycerophospholipid metabolism, arginine biosynthesis, and steroid hormone biosynthesis. IPA network analysis identified AKT, TP53, EGFR, and cAMP as key dysregulated nodes. **Conclusions:** Longitudinal metabolomic profiling demonstrated that COVID-19 vaccination induced distinct but interrelated biochemical changes throughout the vaccination course. The priming dose induced a limited set of early metabolic changes, whereas the booster was associated with more significantly changed metabolites that were involved in lipid, bile acid, steroid, amino acid, and nucleotide pathways. Together, these findings indicate that sequential vaccination is associated with dose-dependent systemic metabolic adaptation, with the booster dose having the largest number of dysregulated metabolites.

## 1. Introduction

The emergence of severe acute respiratory syndrome coronavirus 2 (SARS-CoV-2) in late 2019 resulted in a global pandemic of coronavirus disease 2019 (COVID-19) on an unprecedented scale [1,2]. The World Health Organization (WHO) has recorded over 776 million confirmed cases and more than 7 million deaths worldwide, with the true burden likely to be higher owing to underreporting and missed asymptomatic infections [3,4]. One of the most effective public health interventions to curb the spread of infectious diseases is vaccination [5]. In this regard, COVID-19 vaccines have played a key role in reducing severe disease and death, and in alleviating healthcare system burdens associated with SARS-CoV-2 infection. More than 13.53 billion COVID-19 vaccine doses had been administered globally by August 2024, with approximately 70.6% of the world’s population having received at least one dose [6,7]. 

Vaccines act by presenting antigenic material to the host immune system in a form that primes innate, adaptive, and cellular immune responses without causing disease. This process involves innate immune sensing, antigen processing and presentation, activation of antigen-specific B- and T-cell responses, and subsequent development of immunological memory. Immunity is strengthened through a prime–boost mechanism in which the first dose primes the immune system, and subsequent doses expand and mature memory responses. Multiple vaccine platforms were used during the pandemic, including mRNA-based vaccines, adenoviral vector vaccines, and inactivated whole-virus vaccines, intended to induce recognition of SARS-CoV-2 antigens, particularly the spike protein. mRNA vaccines are nucleic acid-based vaccines encoding antigens that are translated into proteins and can elicit both humoral and cellular immunity. Despite their broad use and overall protective benefit, heterogeneity in vaccine-induced immune responses was observed, as the strength of the immune response is influenced by host factors and the number of administered vaccine doses, raising questions about the molecular and metabolic determinants of vaccination efficacy [8]. Beyond vaccine efficacy and safety, there is growing interest in understanding how vaccines work at the molecular and systems-biology levels [9]. Metabolomics, through the measurement of small-molecule metabolites, offers real-time assessment of perturbations in metabolic pathways [10]. This approach can be used in the context of vaccination to detect alterations in circulating metabolites, thereby providing a systemic view of host biochemical adaptation following immunization. Mass spectrometry (MS)-based metabolomics uses advanced analytical technologies to detect and characterize circulating metabolites. Previous studies have used metabolomics to investigate distinct metabolic phenotypes after vaccination in the context of herpes zoster [11], influenza, and Bacillus Calmette-Guérin (BCG) vaccination, revealing changes in glycolysis, the pentose phosphate pathway, lipid biosynthesis, and central carbon metabolism. 

Recent studies have used metabolomics in COVID-19 research to characterize systemic metabolic disturbances associated with SARS-CoV-2 infection, disease severity, and clinical progression and have shown alterations in amino acid metabolism, the tricarboxylic acid cycle-related metabolites, and lipoprotein or ceramide-associated changes [12,13,14]. Fewer studies have addressed vaccination. Dagla et al. profiled plasma from Pfizer-BioNTech recipients using NMR and LC-MS and reported alterations in amino acid metabolism and in the plasma ceramide and lipid profile [15]. On the other hand, He et al. examined individuals who received an inactivated SARS-CoV-2 vaccine and showed that two-dose vaccination was associated with distinct circulating metabolite changes, identifying glutamic acid as a candidate immune-dependent metabolite biomarker [16]. Lang et al. tracked IgG responses alongside cytokines, lipoproteins, and low-molecular-weight metabolites in individuals receiving two to four COVID-19 vaccine doses and classified participants according to IgG response status [17]. It is known that IgG positivity, however, does not reflect clinical disease status, and in populations with high seroprevalence, the metabolic signature of prior infection cannot be separated from that of vaccination [18]. In our previous study, we identified IgG-specific metabolites that significantly differed between SARS-CoV-2 IgG-positive and IgG-negative individuals [19]. However, the metabolomics changes associated with individual vaccine doses, and their separation from the effects of any prior infection, remain undefined.

In the present study, we carried out an extensive serum metabolomics profiling to characterize dose-related changes in circulating metabolites across the COVID-19 vaccination course, from baseline to after the first and second doses.

## 2. Materials and Methods

### 2.1. Ethical Approval 

In accordance with the Declaration of Helsinki, this study was approved by the Central Institutional Review Board of the Ministry of Health in Riyadh, Saudi Arabia (approval number IRB-21-55M; National Registry Number NCBE-KACST, KSA (H-01-r-009). The SFDA approved the application (application number 21061802). The Public Health Authority (PHA) and the Ministry of Health (MOH) collaborated to establish recruitment centers at the designated primary health centers. Written informed consent was obtained from all individuals. 

### 2.2. Subject Recruitment and Sample Collection

Between October 2021 and July 2022, twenty-nine healthy individuals were selected from a cohort of patients who had received a COVID-19 vaccination (Pfizer, Moderna, and AstraZeneca) for this study. Patients with a history of chronic disease, including type 2 diabetes and hypertension, as well as those not in a fasting state or who had exercised within 60 min prior to sample collection, were excluded. Serum was isolated from whole blood samples collected from various hospitals in Saudi Arabia that participated in a national surveillance effort, and inclusion in this study was based on serologic testing. Specifically, SARS-CoV-2-specific IgG antibodies were measured using Abbott’s SARS-CoV-2 IgG assay (Abbott Laboratories, Abbott Park, IL, USA), which detects IgG antibodies directed against the SARS-CoV-2 nucleocapsid (N) protein. Samples were processed and analyzed according to the manufacturer’s instructions. Because the assay detects antibodies against the nucleocapsid protein rather than the spike protein, a positive result was interpreted as evidence of an immune response associated with previous SARS-CoV-2 infection, rather than as a direct measure of vaccine-induced anti-spike antibodies [1].

Each participant had three serum samples taken: (A) one before the first vaccine dose, (B) the second 20 days after the first dose, and (C) the third 20 days after the second dose. Prior to vaccination, the participants’ SARS-CoV-2 IgG serostatus was evaluated, and all participants were included. 

All participants provided written informed consent and completed a short form to provide additional details, including health conditions, demographics, nationalities, illness severity, and control status. The presence of SARS-CoV-2 IgG antibodies was confirmed by immunoassays.

### 2.3. Sample Preparation

Serum samples for metabolomics were prepared according to a standardized protocol [20,21]. A 50 μL aliquot of serum was combined with 950 μL of a 50% acetonitrile (ACN) in methanol (MeOH) extraction solvent (Fisher Scientific, Ottawa, ON, Canada). Quality control (QC) samples were prepared by mixing equal volumes of 10 μL from each research sample. The mixtures were vortexed in a ThermoMixer (Eppendorf, Hamburg, Germany) at 600 rpm and 25 °C for one hour, then centrifuged at 16,000 rpm and 4 °C for 10 min. The supernatants were collected and evaporated using a Speed-Vac (Martin Christ, Osterode am Harz, Germany). The dried samples were reconstituted in 100 μL of a 50% mobile phase A:B, as detailed in the LC-MS metabolomics section below. 

### 2.4. Metabolomics Analysis

Samples were analyzed using Waters Acquity UPLC and Xevo G2-S QTOF HRMS with an ESI source (Waters, Milford, MA, USA), following previous studies [1,20]. Chromatography used a Waters HSS T3 column (100 × 2.1 mm, 1.8 μm). The gradient from solvent A (0.1% formic acid in water) to B (0.1% formic acid in 50% ACN-MeOH) involved a flow rate of 300 µL/min, with solvent A decreasing from 95% to 5% over 0–16 min, maintained at 5% until 19 min, then returning to 95% at 20 min. MS spectra were acquired in both ESI+ and ESI− modes, with capillary voltages of 3.20 kV and 3.00 kV, respectively. The source temperature was set at 150 °C, desolvation at 500 °C, and gas flows at 800 and 50 L/h. Collision energies ranged from 10–50 V (high) to 0 V (low). Sodium formate calibrated the mass spectrometer in the 50–1200 Da range, using leucine-enkephalin as a reference. The data were collected using Masslynx V4.1, with QC samples randomly inserted to monitor stability, showing RSD% ≤ 30%. 

### 2.5. Statistical Analyses

Raw mass spectrometry data were analyzed utilizing a specified analytical methodology within Progenesis QI software (version 3.0, Waters Technologies, Milford, MA, USA). The process included m/z and retention time peak alignment, peak selection, and deconvolution to ensure data quality and reproducibility. Subsequently, the data underwent median normalization, logarithmic transformation, and Pareto scaling to attain a normal distribution and to reduce heteroscedasticity [21].

Multivariate analyses, encompassing partial least squares discriminant analysis (PLS-DA) and orthogonal PLS-DA (OPLS-DA), were conducted utilizing MetaboAnalyst v.5.0 (McGill University, Montreal, QC, Canada; http://www.metaboanalyst.ca, accessed on 15 May 2025). The efficacy of OPLS-DA models was assessed using R^2^Y (goodness of fit) and Q^2^ (predictive capability), and model resilience was confirmed by 100-fold permutation testing. One-way ANOVA was used to identify significantly altered features, using false discovery rate (FDR) adjusted *p*-values (FDR-*p* < 0.05), as determined by Mass Profiler Professional v.15 (Agilent Technologies, Santa Clara, CA, USA). 

Pathway enrichment and topological studies were conducted to investigate metabolic pathways associated with COVID-19 vaccination-related metabolic alterations. Molecular networks and signaling pathways were analyzed using QIAGEN Ingenuity Pathway Analysis (IPA) version 01-23-01.

### 2.6. Metabolite Identification

The MSI confidence framework guided metabolite identification: substances were confirmed at Level 1 using reference standards with LC-HRMS, based on mass accuracy within 5 ppm, corresponding retention times, and MS/MS spectra. Level 2 involved spectrum library matching and expert review where standards were unavailable. Level 3 classified lipids by their molecular class based on LIPID MAPS, without specifying acyl chain length or double-bond positions. Level 4, lacking diagnostic MS/MS information, was excluded from route analysis. Data integration included HMDB [19], METLIN MS/MS (https://metlin.scripps.edu/landing_page.php?pgcontent=mainPage; accessed 20 May 2025), MassBank, KEGG, LipidMaps, and LipidBlast, using a 5 ppm tolerance and additional validation steps. Exogenous chemicals were manually removed before analysis to focus solely on endogenous metabolic changes. MSI levels are detailed in Supplementary Table S1; biological interpretation is emphasized at Levels 1–2, whereas Level 3 provides class information. Features with high confidence were identified based on precursor masses and spectra, annotated using primary libraries and cross-validation, followed by the manual elimination of xenobiotics [22].

## 3. Results 

### 3.1. Demographics of Participants

This cohort consisted of 29 participants, including 19 males (65.5%) and 10 females (34.5%). The mean age of participants was 36.8 ± 11.4 years. The selected participants had no reported comorbidities or other conditions. Of the 29 participants, 25 had previously been affected by COVID-19, while the remainder had not. 

### 3.2. Metabolomics Profiling

**Figure 1:**
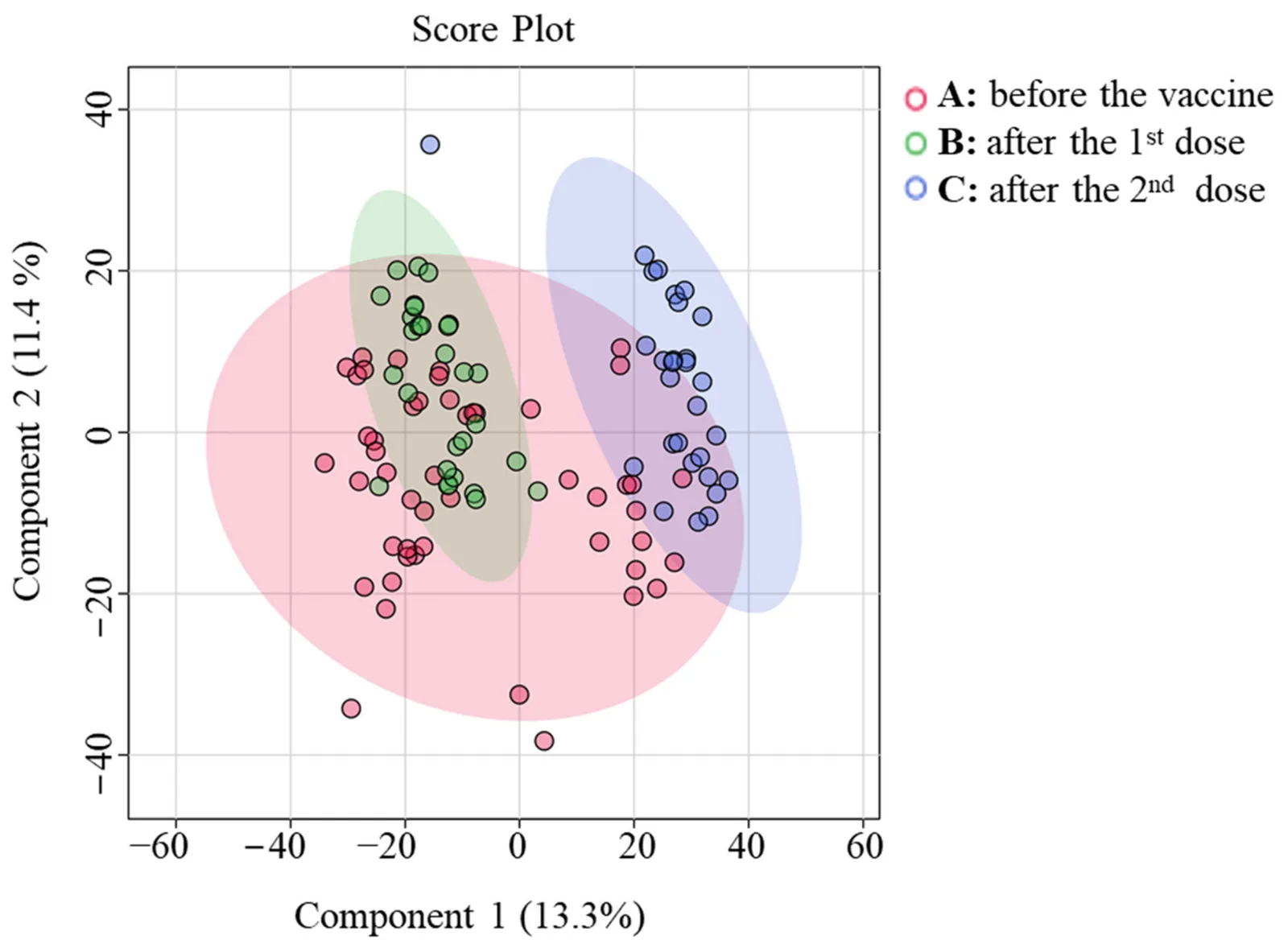
A partial least squares discriminant analysis (PLS-DA) score chart of serum metabolites, comparing serum samples before the vaccine (Group A), after the 1st dose (Group B), and after the 2nd dose (Group C).

LC-HRMS was utilized to assess the metabolomics profile of serum samples. A total of 33,732 mass ion features were detected in both positive and negative ionization modes. Following the application of a frequency filter with a cut-off of 80% of samples in pairs of conditions, 19,517 features remained for further statistical analysis. The metabolic features, previously identified as associated with SARS-CoV-2 IgG serostatus in our earlier study [19] (considered as IgG effect), were matched to the present dataset, and the overlapping metabolites were excluded. This ensured that this study focused solely on metabolites that were driven by the vaccine response without confounding them with those from prior SARS-CoV-2 infections. After exclusion, 19,220 features remained [18]. The PLS-DA score plot showed strong separation between groups B and C. The cumulative variance explained by the first two principal components accounted for 24.7% in the PLS-DA model for the two study groups (PC 1 = 13.3% and PC 2 = 11.4%) (Figure 1).

**Figure 2:**
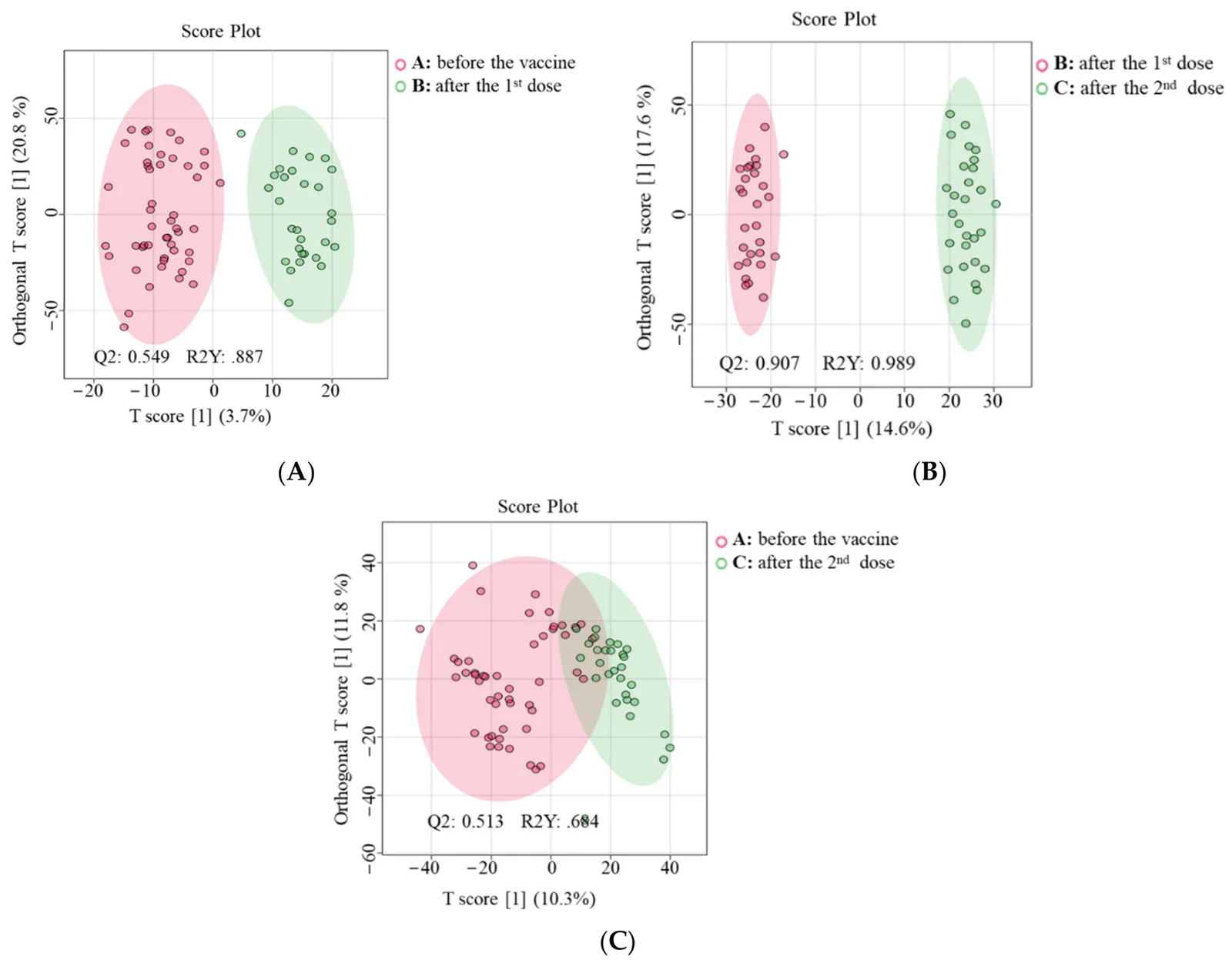
OPLS-DA, orthogonal partial least squares discriminant analysis: Group A, Group B, and Group C. (**A**): Group A vs. Group B (Q2 = 0.549, R2Y = 0.887), (**B**): Group B vs. Group C (Q2 = 0.907, R2Y = 0.989), and (**C**): Group A vs. Group C (Q2 = 0.513, R2Y = 0.684).

OPLS-DA demonstrated a substantial difference between groups A and B, with R^2^Y = 0.887 and Q^2^ = 0.549 (Figure 2A), indicating a significant metabolic disparity between them. Moreover, clear separation was observed between groups B and C with R^2^Y = 0.989 and Q^2^ = 0.907 (Figure 2B). A slight separation was detected between groups A and C with R2Y = 0.684 and Q^2^ = 0.513 (Figure 2C).

One-way ANOVA with Tukey’s post hoc analysis (FDR-adjusted *p* < 0.05) was performed on 19,220 features across the three groups, identifying 6741 significantly dysregulated features. Among these, 1932 were annotated using databases. Of these, 377 metabolites were identified as human endogenous metabolites (Table S1).

### 3.3. Metabolic Pathway and Biomarker Analyses

**Figure 3:**
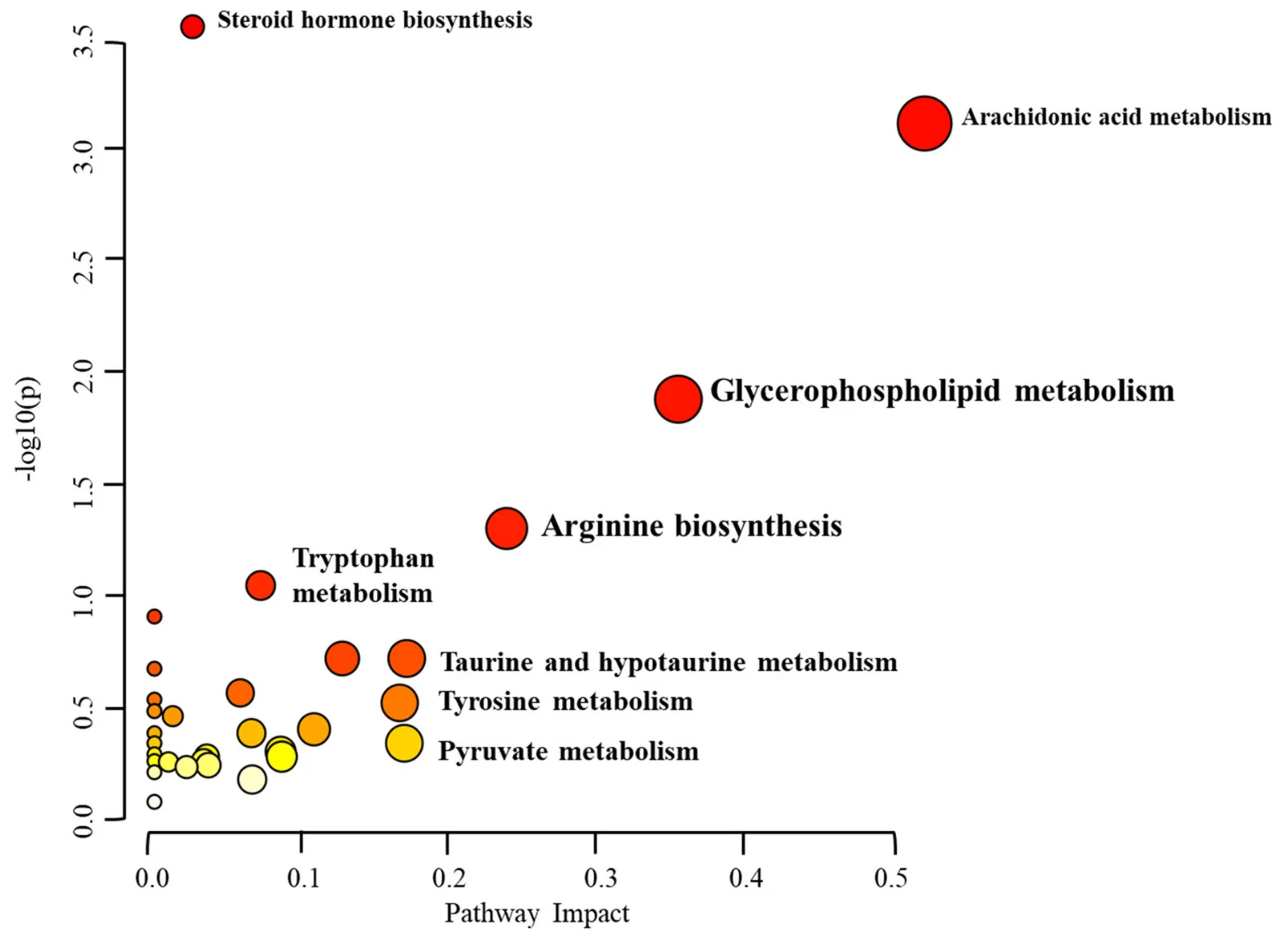
Pathway analysis of significantly dysregulated metabolites (*n* = 377) in the patients compared to pre-dose. The color and size indicate the *p*-value and pathway impact value, respectively. One-Way ANOVA (Tukey’s post hoc, FDR *p* < 0.05).

Pathway analysis of the 377 significantly dysregulated endogenous metabolites indicated that the altered pathways in this cohort were associated with arachidonic acid metabolism, glycerophospholipid metabolism, arginine biosynthesis, and steroid hormone biosynthesis (Figure 3 and Table S2). 

### 3.4. Serum Metabolomics Profiles Change After Each Dose of the COVID-19 Vaccine

Compared with Group A, Group B showed significant dysregulation of 75 human endogenous metabolites (Figure S1A). For Group B versus Group C (second-dose effect), 352 metabolites were significantly dysregulated (Figure S1B). Group A versus Group C (overall vaccine effect) showed 331 significantly dysregulated metabolites (Figure S1C). The complete list is shown in Table S1.

### 3.5. Network Pathway Analysis

Network and pathway analyses were conducted using confidently identified metabolites (MSI Levels 1–2), with MSI Level 3 lipids interpreted only at the class level. Pathway enrichment was assessed via over-representation analysis with FDR correction (q < 0.05), and pathway impact was evaluated using betweenness centrality within the KEGG and Reactome frameworks. Only pathways containing ≥3 MSI Level 1–2 metabolites were considered for inference. Unidentified features were analyzed separately using a Mummichog-based, hypothesis-generating approach.

**Figure 4:**
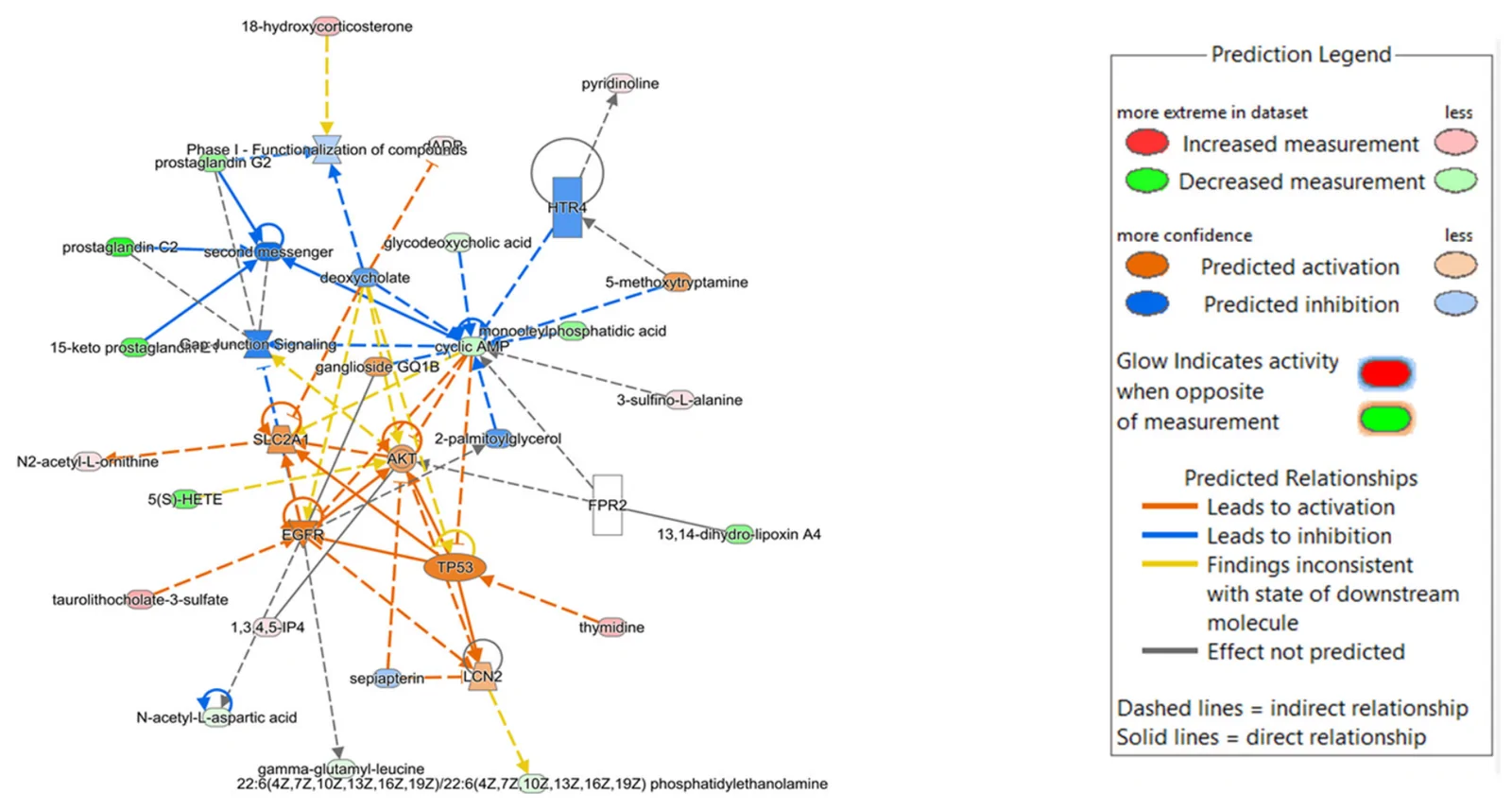
The interaction network for the significantly dysregulated metabolites identified across the vaccination groups (A: pre-vaccination; B: post–first dose; and C: post–second dose). The red and green areas indicate upregulated and downregulated metabolites, respectively. The orange and blue areas indicate activation and suppression by IPA prediction, respectively.

The highest-scoring IPA interaction network is shown in Figure 4. This network contains 19 focus metabolites from our dataset, centered on key signaling regulators, including protein kinase B (AKT), tumor protein p53 (TP53), and the Epidermal Growth Factor Receptor (EGFR), and is functionally related to molecular transport, nucleic acid metabolism, and small-molecule biochemistry. Altered lipid mediators, including prostaglandins and bile acid-related metabolites and cAMP, converge on these hubs, indicating coordinated metabolic and signaling dysregulation. The color coding reflects changes in metabolite abundance and the predicted activation or inhibition of regulatory nodes.

## 4. Discussion

In this study, a longitudinal metabolomics approach was used to characterize the metabolomic profile associated with COVID-19 vaccination in a healthy population. An untargeted metabolomic analysis of serum samples collected before vaccination and 20 days after each dose showed that the systemic biochemical response to COVID-19 vaccination progressed in a dose-dependent manner. Overall, 377 metabolites were quantified at three clinically meaningful time points: (A) pre-vaccination, (B) post-first dose, and (C) post-second dose, enabling three paired comparisons of first-dose effects (A vs. B), second-dose incremental effects (B vs. C), and overall vaccine effects (A vs. C). Moreover, metabolites associated with SARS-CoV-2 IgG seropositivity were excluded, allowing the observed systemic responses to be attributed to vaccine-induced effects rather than to prior infection. This approach was designed to overcome a limitation of previous metabolomics studies on COVID-19 infection and vaccination, in which infection- and vaccine-induced metabolic effects overlapped. The metabolites identified in our study generally belonged to the categories of eicosanoids/oxylipins, glycerophospholipids/sphingolipids, glycerolipids/fatty acids, bile acids, amino acids, nucleotides, and tryptophan/kynurenine pathway intermediates. 

### 4.1. Changes in Metabolites Across the Vaccination Course: Priming and Booster Phase Responses (A vs. B, A vs. C, and B vs. C)

We identified a broad directional shift across the different metabolite categories over the course of COVID-19 vaccination. Of the 377 metabolites profiled, 59 were significantly dysregulated in common across the three comparisons. A biphasic pattern was noted for 47 metabolites that were downregulated in the first comparison (A vs. B), followed by upregulation in both the second (B vs. C) and third (A vs. C) comparisons. These included metabolites belonging to the eicosanoid, prostaglandin, glycerolipid, phospholipid, steroid glucuronide, and mono- and diacylglycerol classes. We observed a significant decrease in the levels of PGG2, 5,6-dihydroxyprostaglandin, 20-trihydroxy-leukotriene-B4, 5-HETE, and 13,14-dihydro-PGF2α. The identified eicosanoids, including PGG2, are intermediates of the cyclooxygenase (COX) pathway, which serves as the gateway to the prostanoid cascade, with downstream conversion to PGH2 and other prostaglandins [23]. The increase in the levels of these metabolites after the first dose (i.e., the priming phase), followed by a decrease after the second dose, has been proposed to reflect modulation of the COX-arachidonic acid axis [24]. In addition, bile acids, such as bisnorcholic acid and 3-Sulfodeoxycholic acid, along with steroid conjugates, including 11-oxo-androsterone glucuronide and 4-hydroxyandrostenedione glucuronide, were also noted to follow the same biphasic pattern. Previous studies have noted that bile acids and steroid hormones are involved in regulating immune homeostasis [25,26]. 

On the other hand, eleven metabolites demonstrated an inverse pattern, i.e., decreased after the first dose and then increased subsequently. These included citrulline, nicotinic acid ribonucleoside, and carnitines. In the context of immunity, citrulline, a by-product of arginine metabolism, is required for the development of innate, adaptive, and cellular immune responses through the arginine–citrulline–nitric oxide pathway [27]. Alterations in arginine-associated metabolites often correlate with immune response activation by increasing amino acid uptake and utilization to promote cell proliferation [28,29,30]. Another notable metabolite was nicotinic acid ribonucleoside, a precursor to nicotinamide adenine dinucleotide (NAD), a cofactor for several immune regulatory enzymes, including CD38. Immune cells, including B cells, T cells, and macrophages, carry CD38, which utilizes NAD^+^ during immune cell activation, differentiation, and cytokine release [31]. As CD38 expression increases following immune cell activation, an initial increase followed by a decrease in this precursor may reflect progressive consumption of NAD+ biosynthetic intermediates across the vaccination course [32].

### 4.2. Changes Observed in Metabolites in Priming Phase Response (A vs. B)

We identified the dysregulation of 14 unique metabolites during the priming phase (A vs. B). Among these, L-3-hydroxykynurenine (3-HK) was identified uniquely in this phase, while four metabolites were shared with the booster phase (B vs. C), and nine with the overall vaccine response (A vs. C). 3-HK is a downstream catabolite of tryptophan metabolism produced via the kynurenine pathway and was found to be increased in individuals receiving the first dose compared with pre-vaccinated individuals. Tryptophan metabolism is known to be altered post-vaccination. The activation of the tryptophan–kynurenine pathway in antigen-presenting cells has previously been shown to control the proliferation and response of T cells []; the increase in 3-HK found in the current study is indicative of activation of this pathway, though not enough evidence exists to show this regulation was taking place in the current study population. This early increase in 3-HK activity is also considered a predictor of a strong humoral response [33]; even though antibody titers were not analyzed in this particular cohort, this relationship remained unvalidated. Previous targeted and untargeted metabolomics studies have similarly reported alterations in the kynurenine pathway influenced by COVID-19 infection [34]. The increase in levels of 3-HK was transient and was not sustained through to the booster or full vaccination course, suggesting an accelerated tryptophan catabolism was part of an early immune rerouting of metabolic resources. 

Additionally, eight metabolites were significantly dysregulated in common with the second comparison (B vs. C). Notable among these was arachidonic acid, a precursor to prostaglandins, thromboxane, and leukotrienes, which increased after the first dose. These bioactive lipids are well-known mediators of inflammation that influence immune cell regulation and function [35]. Their increase may indicate early immune activation in response to initial antigen exposure. We also noted an early increase in 5-Androstenediol, a steroid precursor with immunomodulatory properties. Androgens are known to shape immune cell behavior, and their rise during priming may reflect a transient neuroendocrine- or steroid-related host adaptation that subsequently declines after the booster. These findings align with Liu et al., who observed a similar pattern of metabolic shifts during the early vaccination period [33].

### 4.3. Changes Observed in Metabolites in Booster Phase Effect (B vs. C)

In the present study, the comparison between the first and second vaccine doses (B vs. C) revealed significant differential regulation in 34 unique metabolites. An increase was observed in 26 metabolites, including oxylipin- and prostaglandin-linked phospholipids and lysophospholipids, and several LysoPA species, as well as DL-Dopa, 3-Methoxytyrosine, 2-Methoxyestrone, and 3-glucuronide in the priming dose compared to the booster. The shifts in these metabolites reflect a distinct metabolic alteration between the primary and booster doses. These changes are consistent with the difference in metabolism between the primary and booster doses, which corresponds with the observations that vaccination affects phospholipids and lysolipids [15,16,33,36]. Specifically, the increase in LysoPA species is significant, as lysophosphatidic acid is a recognized bioactive lipid that modulates immune-cell behavior, including CD8^+^ T-cell metabolic fitness and broader immune regulation [37]. Two additional metabolites of interest, DL-Dopa and 3-methoxytyrosine, were increased after the first dose relative to the second. Dopamine-related pathways have been increasingly recognized as immunomodulatory, with reported effects on immune-cell activation and inflammatory signaling [38,39,40]. 

On the other hand, we observed a significant decrease in seven metabolites, including phospholipids, myo-Inositol 1,3,4,5-tetrakisphosphate, and 3-Hydroxyheptanoic acid between the primary and booster doses. Among these, myo-inositol 1,3,4,5-tetrakisphosphate (IP4) was particularly notable, as it serves as a co-activator for B-cell synthesis and survival, as well as for immune-cell signaling and migration. It has previously been shown that increased IP4 affects the regulation of the ERK pathway in B cells [41]; however, we cannot determine if this pathway was activated in our subjects based on metabolite levels alone. Phosphoinositide signaling more broadly controls polarization, protrusion, and motility in immune cells [40].

### 4.4. Changes Observed in Metabolites in Vaccine Effect (A vs. C)

We identified 18 metabolites that were significantly differentially regulated in the vaccine effect (A vs. C) group, including membrane lipids, bile acid derivatives, nucleotides, inositol lipid signaling, and fatty-acid oxidation-related metabolites. Notable among these was a decrease in the level of cyclic AMP in the pre-vaccination group compared to the overall vaccination group. Cyclic AMP is a known central regulator of immune cell function that suppresses effector T cell activation, inhibits NF-κB signaling, and promotes regulatory T cell induction [42]. Elevation of cAMP following immunization is in line with, but does not prove, activation of this regulatory pathway in our participants. The cAMP-PKA signaling pathway in T cells is initiated through the binding of PGE2 and other extracellular ligands, such as catecholamines and adenosine, which was also identified in our study.

Bile acid derivatives, deoxycholic acid 3-glucuronide and cholylserine, were also noted to be dysregulated, further suggesting a persistent remodeling of bile acid metabolism and conjugation, consistent with reports that bile acids act as immune-active signaling molecules through receptors such as FXR and TGR5, with documented effects on macrophages, dendritic cells, NK cells, and T cells [43]. Our findings are in line with Alexander et al., who demonstrated that specific bile acid species were inversely associated with COVID-19 vaccine antibody responses in IBD patients [44], and with the COVID-19 vaccine metabolomics literature, showing that bile acid pathways can be altered after vaccination [33]. The lipid species identified in this set point to ongoing glycerophospholipid- and oxylipin-linked membrane remodeling, suggesting that the overall vaccine effect included sustained reorganization of phospholipid pools and inflammatory lipid mediator handling. In particular, the LTE4-containing PE species is notable because leukotriene E4 is an established immune-active lipid mediator, implying that elements of leukotriene-linked signaling remained part of the post-vaccination metabolic landscape [45]. 

### 4.5. Metabolic Changes Shared Across the Vaccination Course

Our data revealed that the largest number of metabolites, consisting of 250 metabolites, was shared between the overall vaccine (A vs. C) and the booster groups (B vs. C) comparison, representing the cumulative signature after receiving both doses of vaccination. The majority of metabolites within this group belonged to membrane and signaling lipids, followed by eicosanoids, amino acids and their derivatives, bile acids and their conjugates, nucleotides, and cardiolipin classes. Most metabolite classes showed mixed directionality, whereas cardiolipins were increased and the eicosanoids were observed to be consistently decreased at C. The mixed directionality of the metabolites may reflect selective lipid and metabolic remodeling that is a feature of immune-cell activation, antigen presentation, and immune-cell communication. Membrane lipids are known to regulate T-cell signaling by shaping lipid domains, immune synapse formation, protein recruitment, and receptor mobility, making this lipid class highly relevant to vaccine-induced immune activation. Therefore, the mixed direction observed in these lipid species may reflect reorganization of membrane architecture and lipid-derived signaling as part of immune-cell regulation and signaling [46,47,48,49]. 

On the other hand, cardiolipins, which are mitochondrial metabolites involved in supporting mitochondrial structure, respiratory-chain organization, and cellular bioenergetics, were increased after the full vaccination course. Mitochondrial adaptation is known to be essential for antigen-driven activation and differentiation, for effective CD8^+^ T-cell responses, memory T-cell development, and metabolic adaptation during immune activation [50]. Previous studies have shown that lipid-related metabolites, phospholipids, and circulating eicosanoid networks were altered post-vaccination [33,51,52]. The changes in the other identified metabolites are detailed in Table S3.

### 4.6. Metabolic Network Remodeling and Immune Signaling Following Vaccination

Network pathway analysis suggested that the significantly differentially altered metabolites identified in our study were linked to broader host–response signaling pathways. A total of 19 metabolites centered around AKT, EGFR, and TP53, indicating their connectedness to pathways linking metabolic state to immune-cell regulation and activation, survival, and inflammatory signaling associated with vaccination, providing evidence of the biological significance of the altered metabolites. 

However, several pathways that were discovered through this research, including glycerophospholipid metabolism, eicosanoid metabolism, amino acid metabolism, and bile acid metabolism, have also been found through metabolomics studies on vaccines for influenza, herpes zoster, and malaria [53,54,55]. Thus, the pathways described above might represent some conserved metabolic characteristics of vaccines’ actions rather than being specific to the SARS-CoV-2 vaccination. However, differences between vaccines’ antigens and platforms, sampling procedures, populations of subjects included in the studies, and techniques used in analyses make comparisons difficult. Thus, despite the fact that the results of this study are consistent with the general trend of vaccine-induced metabolic changes, it cannot be concluded as to which of the metabolites described in this research are specific to coronavirus disease 2019 vaccination and which are universal ones.

Our study has a number of limitations. First, because this retrospective cohort was derived from the Ministry of Health national vaccination program, the vaccine platform was determined by program availability rather than by study allocation. The specific vaccine platform received by each participant is not consistent, suggesting that the observed metabolic changes reflect immune-metabolic responses to COVID-19 vaccination regardless of the type of vaccine. The inclusion of mRNA- and adenoviral-vector vaccines may, therefore, have introduced biological heterogeneity and residual confounding. Furthermore, the small number of participants within the Moderna, Pfizer, and AstraZeneca vaccine subgroups limits the statistical power of vaccine-specific subgroup analyses. Therefore, any differences observed among vaccine types should be considered exploratory and interpreted with caution. Secondly, as this was a retrospective cohort, vaccine-specific antibody titres were not available and, therefore, the magnitude of the observed metabolic alterations could not be directly correlated with the strength of the humoral immune response. The reported metabolites should be interpreted as exploratory vaccination-associated signals across the overall cohort rather than as vaccine-brand-specific biomarkers or evidence of equivalent responses among vaccine platforms. Our sample size was also small and, therefore, the findings should be considered exploratory, requiring validation in future studies with larger cohorts and documented vaccine type to validate our findings and determine platform-specific metabolic signatures.

## 5. Conclusions

In conclusion, longitudinal serum metabolomic profiling revealed that COVID-19 vaccination induces structured, dose-dependent biochemical remodeling throughout the vaccination course. The priming dose induced a limited set of early metabolic changes, whereas the booster was associated with more significantly changed metabolites that were involved in lipid, bile acid, steroid, amino acid, and nucleotide pathways. Shared metabolites showed that some metabolic modifications were sustained after the priming dose, whereas other modifications occurred after the booster dose. Overall, these findings indicate that sequential COVID-19 vaccination is associated with systemic metabolic adaptation and support the value of metabolomics in improving understanding of the host biochemical response to vaccination.

## Data Availability

The original contributions presented in this study are included in the article/Supplementary Materials. Further inquiries can be directed to the corresponding authors.

## Supplementary Materials

The following supporting information can be downloaded at: https://www.mdpi.com/article/10.3390/vaccines14090771/s1, Table S1: Endogenous metabolites; Table S2: Pathway analysis; Table S3: Classification and directionality of the 250 shared metabolites. Figure S1: Serum metabolomic profiles across the COVID-19 vaccination timeline, comparing baseline (pre-vaccination), post–first dose, and post–second dose states. (**A**) Profile of 75 significant metabolites that were dysregulated in the 1st dose effect of A vs. B (Table S1). (**B**) Profile of 352 significant metabolites that were dysregulated by the 2nd dose effect of B vs. C (Table S1). (**C**) Profile of 331 significant metabolites that were dysregulated by the vaccine effect of A vs. C (Table S1).
