# Supplementary material for: Metabolic Signatures Associated with COVID-19 Vaccination in Serum from Healthy Individuals
Source: Vaccines (Basel). 2026 Sep 2;14(9):771. doi: 10.3390/vaccines14090771 (PMC13611412; doi:10.3390/vaccines14090771)
Supplement: Supplementary file 1 [file vaccines-14-00771-s001.zip › Figure S1.pdf]

(A)

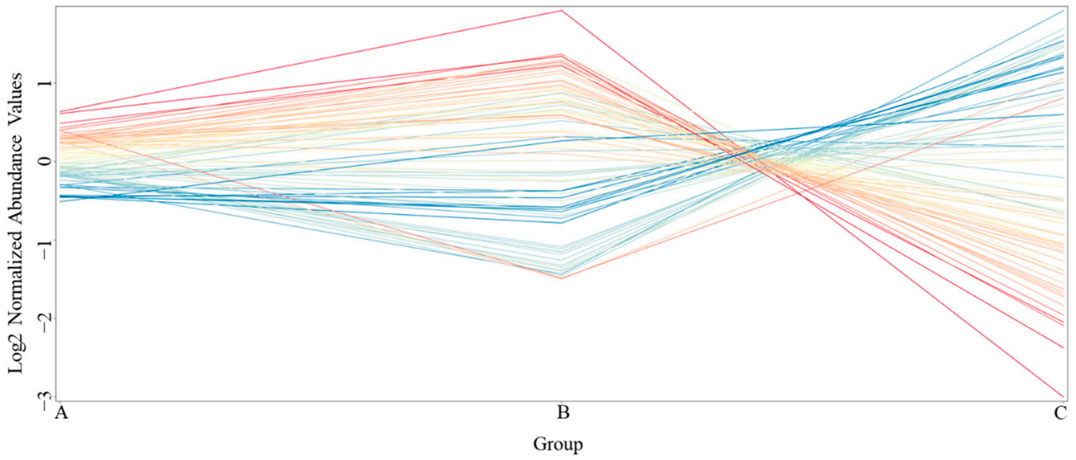
Log2 Normalized Abundance Values
1
0
−1
−2
−3
A
B
C
Group

(B)

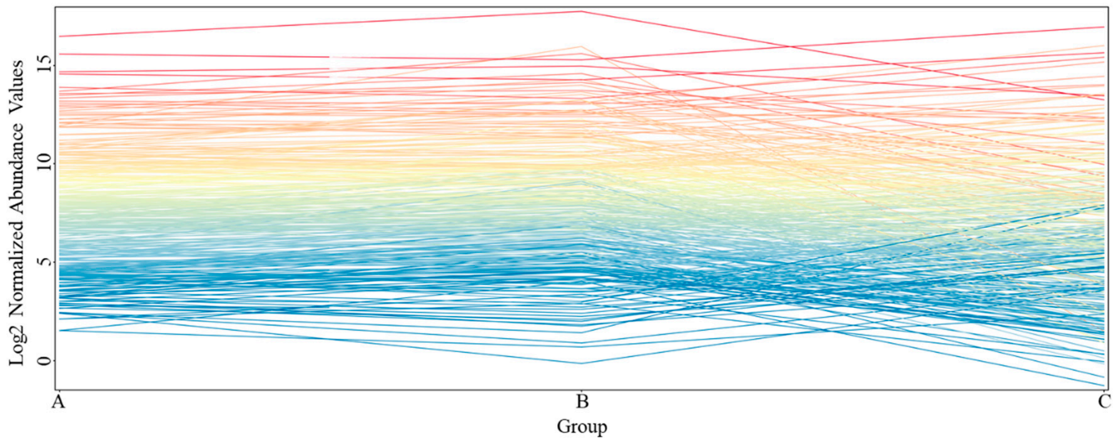
Log2 Normalized Abundance Values
15
10
5
0
A
B
C
Group

(C)

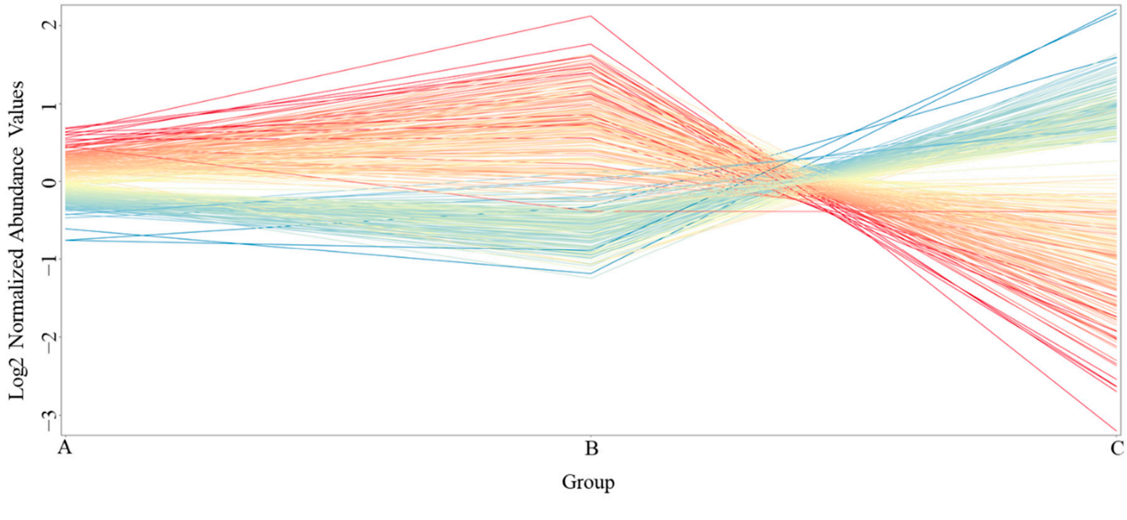
Log2 Normalized Abundance Values
2
1
0
−1
−2
−3
A
B
C
Group

Figure S1. Serum metabolomic profiles across the COVID-19 vaccination timeline, comparing baseline (pre-vaccination), post–first dose, and post–second dose states. (**A**) Profile of 75 significant metabolites that were dysregulated in the 1st dose effect of A vs. B (Table S1). (**B**) Profile of 352 significant metabolites that were dysregulated by the 2nd dose effect of B vs. C (Table S1). (**C**) Profile of 331 significant metabolites that were dysregulated by the vaccine effect of A vs. C (Table S1)..
